# Complete Genome Sequences of Two Plant Growth-Inhibiting Bacteria, Acinetobacter ursingii M3 and Asticcacaulis excentricus M6, Isolated from Duckweed (Lemna minor)

**DOI:** 10.1128/MRA.01092-18

**Published:** 2018-09-27

**Authors:** Hidehiro Ishizawa, Masashi Kuroda, Daisuke Inoue, Michihiko Ike

**Affiliations:** a Division of Sustainable Energy and Environmental Engineering, Graduate School of Engineering, Osaka University, Suita, Osaka, Japan; University of California, Riverside

## Abstract

Acinetobacter ursingii M3 and Asticcacaulis excentricus M6 are plant growth-inhibiting bacteria that reduce the yield of the duckweed Lemna minor. We report here the complete genome sequences of A. ursingii M3 and A. excentricus M6, sequenced using the PacBio RS II platform.

## ANNOUNCEMENT

Plant growth-inhibiting bacteria (PGIB), also called deleterious rhizobacteria, repress host growth without any disease symptoms other than reduced growth (1). Despite their ubiquitous occurrence in the plant rhizosphere and potential negative influence on crop productivity (2–4), the molecular aspects of PGIB and their plant interactions are not well understood.

We report the genome sequences of Acinetobacter ursingii M3 and Asticcacaulis excentricus M6, the PGIB strains of the duckweed Lemna minor. Previously, 22 distinct bacterial strains were isolated from L. minor RDSC 5512 using conventional culture methods, and these were separately cocultured with sterilized Lemna minor samples to examine the effect on host growth. Unlike the majority of the isolates, which had positive or neutral effects on the host, A. ursingii M3 and A. excentricus M6 decreased the weekly yield of duckweed by 10 to 20% (4). Plant growth inhibition by A. ursingii M3 and A. excentricus M6 is reportedly accompanied by the enhanced accumulation of reactive oxygen species and stimulation of antioxidant enzymes in plant cells (5). Since duckweed is an emerging crop that can be cultured with wastewater and yield high-value biomass (6), attention has been directed at improving its productivity by considering plant-microbe interactions. For that purpose, the mechanisms by which these PGIB reduce host growth need to be well understood.

The genomic DNA of A. ursingii M3 and A. excentricus M6 was extracted using the illustra bacterial genomicPrep mini spin kit (GE Healthcare, Little Chalfont, UK) according to the manufacturer’s protocol. Sequencing was performed with a PacBio RS II platform (Pacific Biosciences, Menlo Park, CA, USA) using a single-molecule real-time (SMRT) cell 8Pac version 3 and a DNA polymerase binding P6 kit (Pacific Biosciences). Approximately 12 µg of DNA was used for construction of a SMRT cell library. For the genomes of A. ursingii M3 and A. excentricus M6, we obtained 107,291 and 152,454 quality-filtered subreads (*N*_50_, 15,204 and 12,763 bp, respectively), totaling 1,126 and 1,373 Mb, respectively. *De novo* assembly was performed using the Hierarchical Genome Assembly Process (HGAP) version 3.0 with default settings. Gene prediction and annotation were conducted with Rapid Annotations using Subsystems Technology (RAST; see http://rast.nmpdr.org/).

The genome assembly yielded three circular contigs each for A. ursingii M3 and A. excentricus M6. Table 1 summarizes the genome statistics. It was found that the chromosome of A. ursingii M3 contained seven sets of 5S-23S-16S rRNA genes, while A. excentricus M6 has one and two sets of 5S-23S-16S rRNA genes in chromosome 1 and chromosome 2, respectively. Further investigation is needed to screen for candidate genes involved in their plant growth inhibition. To our best knowledge, this is the first report of a PGIB genome sequence.

**TABLE 1:** Genome information for Acinetobacter ursingii M3 and Asticcacaulis excentricus M6

Strain	Replicon	Coverage depth (fold)	Genome size (bp)	G+C content (%)	No. of coding sequences	GenBank accession no.
A. ursingii M3	Chromosome	251	3,429,552	40.2	3,230	AP018824
	Plasmid pAURM-1	175	105,528	41.0	114	AP018825
	Plasmid pAURM-2	206	33,916	38.4	37	AP018826
A. excentricus M6	Chromosome 1	277	2,422,803	60.7	2,157	AP018827
	Chromosome 2	254	1,079,025	60.9	950	AP018828
	Plasmid pASEM-1	368	291,367	59.3	216	AP018829

### Data availability.

The genome sequences of A. ursingii M3 and A. excentricus M6 have been deposited at DDBJ/EMBL/GenBank under the accession numbers AP018824 to AP018826 (BioProject PRJDB7166) and AP018827 to AP018829 (BioProject PRJDB7167), respectively.
